# Understanding the relationship between social media use for information acquisition and life satisfaction from a knowledge, beliefs, and practices perspective

**DOI:** 10.3389/fpsyg.2025.1678675

**Published:** 2025-10-14

**Authors:** Mengru Sun

**Affiliations:** College of Media and International Culture, International Communication Institute, Zhejiang University, Hangzhou, China

**Keywords:** social media, information acquisition, knowledge, beliefs, practices theory, life satisfaction, health knowledge, self-efficacy

## Abstract

**Background:**

The relationship between social media use and subjective well-being is an important research topic. Nevertheless, limited research has specifically explored the role of social media use for information acquisition on life satisfaction. This study investigated how the use of social media for information acquisition influenced life satisfaction by examining a proposed theoretical model.

**Methods:**

Data were collected online through a survey company. A total of 1,651 individuals responded to the survey invitation via email and participated in the study. Data cleaning was conducted, resulting in a final valid sample of 1,513 cases. In this study, SPSS 22.0 was used to perform descriptive statistics and correlation analysis on the data. The mediation model was tested using the SPSS macro PROCESS (Model 6), with the significance of indirect effects assessed through the bootstrap method.

**Results:**

The results indicated that social media use for information acquisition exerted both a direct positive effect on life satisfaction and an indirect effect through the sequential mediation of health knowledge and self-efficacy. Notably, only perceived knowledge demonstrated a significant mediating effect, whereas actual knowledge did not. Additionally, self-efficacy significantly mediated the relationship in conjunction with both types of knowledge.

**Conclusion:**

By differentiating the roles of perceived and actual knowledge within digital environments, this study extends of the knowledge, beliefs, and practices theory and provides practical implications for health-related interventions.

## Introduction

As social media has become an integral part of daily life, its influence on users spans various psychological and behavioral dimensions. Scholars have identified multiple motivations for social media use, including social interaction, self-presentation, information seeking, and entertainment (Bal and Bicen, 2017; Chen and Wang, 2021; Hollenbaugh and Ferris, 2014; Yilmaz et al., 2025). Previous research has predominantly examined the effects of social media use for social purposes on users’ subjective and psychological well-being (e.g., Pang, 2019). To explain this relationship, several mechanisms have been proposed, such as social capital, social support, perceived connectedness, and social integration (Luo and Hancock, 2020).

However, limited research has examined the psychological effects of other motivations for social media use. As social media engagement grows, there is an increasing tendency for individuals to rely on these platforms for information acquisition (Chen and Wang, 2021). For example, in the United States, more than 54% of Americans regularly consume news via social media (Pew Research Center, 2024). Similarly, in China, 77.25% of people access news information through WeChat groups, underscoring the role of social media as a primary news source (Chinese Academy of Social Sciences, 2021). Notably, during the past COVID-19 pandemic, despite the prevalence of unverified health-related content on social media, many users turned to these platforms to obtain and assess relevant information. Such behavior suggests that the use of social media for information acquisition may influence users’ life satisfaction.

The World Health Organization (2012) defines quality of life as a multidimensional concept encompassing social, psychological, and physical well-being. Previous research using the knowledge, attitudes/beliefs, and practices (KAP) theory has predominantly focused on behavioral health outcomes, such as physical activity (Van Vuuren et al., 2021; Zahiruddin et al., 2018). However, both social and psychological well-being are essential dimensions of health. Furthermore, over the past several decades, life satisfaction has received extensive attention from sociologists and psychologists as a key indicator for assessing quality of life at both individual and societal levels. It serves as a valuable tool for monitoring social change and informing public policy improvements (Graça and Brandão, 2024; Jaspal, 2024).

Therefore, this study seeks to extend the KAP framework into the domain of subjective well-being to examine how social media use for information acquisition influences individuals’ life satisfaction. Previous studies have suggested that, in the digital context, the sheer time spent on social media has limited direct impact on psychological and social outcomes (Weiser, 2001). Rather, the nature of usage behaviors plays a critical role. For instance, research indicates that social media use motivated by information acquisition and social needs is positively associated with offline social capital and life satisfaction (Kim and Kim, 2017). Specifically, information seeking on social networking sites has been identified as a significant positive predictor of social capital (Gil de Zúñiga et al., 2012). In contrast, use driven by entertainment and recreational needs shows no significant effect on social capital and may even reduce psychological well-being (Guo et al., 2014). Furthermore, both health knowledge and self-efficacy have been consistently identified in previous literature as robust predictors of life satisfaction (Döş, 2023; Suh et al., 2012). Guided by the KAP model, this study investigated the relationships between social media use for information acquisition and the variables of health knowledge, self-efficacy, and life satisfaction. Data were collected through an online survey administered via Sojump, one of China’s largest professional survey platforms. It is anticipated that the findings not only broaden the applicability of the KAP theory but also offer practical insights for enhancing individual life satisfaction.

## Social media use for information acquisition and life satisfaction

### Relevant theoretical background

Communication researchers have increasingly focused on the effects of social media use on life satisfaction, recognizing the vital role of psychological well-being in individuals’ lives. As a cognitive dimension of subjective well-being, life satisfaction refers to an individual’s subjective evaluation of their quality of life based on personal standards (Diener, 1996). Research has documented numerous benefits associated with higher life satisfaction, including improved physical and mental health, positive social relationships, and greater educational and occupational success (Steel et al., 2019; Willroth et al., 2020). Moreover, recent longitudinal studies indicate that high life satisfaction may buffer the impact of stressful life events (Suldo and Huebner, 2004), highlighting the importance of identifying early factors that contribute to life satisfaction. Despite extensive research on determinants such as socioeconomic status and health education, relatively few studies have explored the growing influence of social media use for information acquisition in shaping life satisfaction (Lee et al., 2014).

In other words, the impact of social media use on individual psychological development remains incompletely understood, and its precise relationship with life satisfaction continues to be unclear. Some studies suggest that social media use positively influences psychological development by facilitating pleasurable experiences (Blomfield Neira and Barber, 2014; Valkenburg et al., 2006) and enhancing life satisfaction (Guo et al., 2014). In contrast, other research points to a negative correlation between social media use and life satisfaction, indicating potential detrimental effects on health and well-being (Hinsch and Sheldon, 2013). These mixed findings underscore the need to further explore the underlying mechanisms through which social media use influences users’ psychological outcomes.

### Research hypotheses

We propose that the contingent effects of social media may be attributable to the diversity of user motivations, which can exert varying influences on subjective well-being. The purpose of social media use refers to how and why individuals engage with these platforms. These motivations and gratifications are shaped by personal psychological characteristics and needs, resulting in divergent outcomes and impacts (Lim, 2023). Although motivations for social media use are multifaceted, they can be broadly categorized into several types, including entertainment, information seeking, social interaction, self-expression, passing time, professional advancement, and following new trends (Hollenbaugh and Ferris, 2014; Jung et al., 2007). Previous research has predominantly examined social and communicative uses of social media—such as self-disclosure and instant messaging—and generally reports positive effects on life satisfaction (Chan, 2015; Hawi and Samaha, 2017; Valkenburg et al., 2022). For instance, perceived connectivity, social support, and social capital have been identified as key mediators between social media use and life satisfaction (Pang, 2019).

Information acquisition represents another primary motivation for social media use (O'Brien et al., 2017). Social media facilitates easier access to and sharing of information across diverse platforms. In the health domain, for instance, users frequently engage in both acquiring and disseminating health-related information (Wei and Gao, 2017). Health information sharing behavior is defined as “the purposive transmission of health information to others”—a form of supportive communication aimed at benefiting others and strengthening social relationships (Liu et al., 2019, p. 1825). Similarly, information acquisition encompasses both active seeking and passive encountering or scanning of health information (Shi et al., 2013). Social media platforms not only enhance the convenience and personalization of information dissemination but also blur the traditional boundaries between information producers and consumers. By delivering tailored content, these platforms enable users to explore and cultivate new interests, thereby facilitating truly interactive communication. This immediate and participatory nature of information behavior may contribute positively to users’ life satisfaction (Wei and Gao, 2017). Nonetheless, the underlying mechanism linking social media-based information acquisition to life satisfaction remains underexplored. Therefore, we propose the following hypothesis:

*Hypothesis 1*: Social media use for information acquisition is positively related to life satisfaction.

## The role of health knowledge and self-efficacy

Previous studies have indicated that self-efficacy and knowledge serve as significant predictors of health outcomes, including preventive behaviors and mental well-being (Yıldırım and Güler, 2022). Furthermore, as posited by the knowledge, attitudes, and practices (KAP) framework, knowledge and beliefs can sequentially influence practices (Qu et al., 2021). In line with this theoretical proposition, the present study examines the mediating roles of health knowledge and self-efficacy within the relationship between social media use for information acquisition and life satisfaction.

### The mediating role of health knowledge

Knowledge constitutes a fundamental component of the decision-making process and serves as a key factor in shaping treatment choices and facilitating active involvement in shared decision-making. Health knowledge refers to factual understanding encompassing basic physical, psychological, and social aspects of health, along with awareness of common misconceptions (Beier and Ackerman, 2003). It has been widely recognized as a critical determinant of health behaviors (Noar and Zimmerman, 2005; de Melo Ghisi et al., 2014). For instance, numerous studies have demonstrated that knowledge of diabetes can predict adherence to related self-management behaviors (Chan and Molassiotis, 1999; Piccinino et al., 2015). Furthermore, limited knowledge about aging has been shown to negatively affect various dimensions of quality of life, including psychological well-being (Suh et al., 2012). This emphasized that a proper understanding of the aging process requires adequate knowledge of aging. Their findings indicated that greater knowledge about aging among older adults is associated with higher life satisfaction, as such knowledge helps clarify misconceptions and promote realistic expectations about aging.

Prior research has further distinguished between actual knowledge (objective knowledge) and perceived knowledge (subjective knowledge), noting their differential impacts on health outcomes (Rimal, 2000). Over the past decades, providing information about diseases and treatments has been a systematic approach widely adopted in health education, becoming a recognized necessity to help patients and their families better understand and manage chronic conditions (Chow, 2017). Without adequate understanding of the disease and the insights derived from such knowledge, improvements in coping strategies are likely to remain suboptimal (Chow, 2017). Although health education is considered a fundamental method for raising patient awareness, evidence suggests that its overall effectiveness may fall short of expectations (Nutbeam, 2000). This limited impact may be attributable to the distinct roles played by actual versus perceived knowledge (Rimal, 2000). While several studies have investigated the effects of interventions on patient well-being, relatively few have explicitly examined how different types of health knowledge predict life satisfaction.

Studies have also indicated that perceived knowledge, rather than actual knowledge, serves as a significant predictor of self-efficacy (Martinussen et al., 2015; Torres and Marks, 2009). These findings further suggest that understanding how to achieve behavioral goals is a necessary yet insufficient condition for successful behavioral implementation. Additional research has corroborated that knowledge offers limited explanatory power compared to other determinants of healthy behavior. For instance, Hale and Trumbetta (1996) highlighted the superior importance of self-efficacy over knowledge, demonstrating that knowledge does not significantly predict behavioral risks related to sexually transmitted diseases among women. In other words, health knowledge may exert its influence on health outcomes primarily through the enhancement of self-efficacy. Within the framework of social support theory, informational knowledge constitutes a key dimension of social support (Smith-Frigerio, 2021). Given these insights, it remains essential to further clarify whether actual knowledge and perceived knowledge play distinct roles in mediating the relationship between social media use for information acquisition and individuals’ life satisfaction.

### The mediating role of self-efficacy

Another variable that has received relatively extensive scholarly attention is self-efficacy and its relationship to health outcomes. Self-efficacy refers to an individual’s subjective assessment of their own capabilities when confronting challenges and setbacks. Bandura (1997) defined it as the “belief in one’s capabilities to organize and execute the courses of action required to produce given attainments” (p. 3). The development of self-efficacy often begins with the acquisition of sufficient information regarding a specific behavior. Moreover, self-efficacy is considered a crucial link between knowledge and behavior, enabling individuals to confidently make informed decisions that help prevent health-related issues (Hall et al., 2016). It encompasses behavioral skills such as effectively communicating with healthcare professionals and overcoming barriers to accessing health services (Ye, 2010).

Concurrently, the role of self-efficacy as a determinant of psychosocial adaptability has garnered increasing scholarly attention in relation to health behaviors. Rimal (2000) demonstrated that self-efficacy not only exerts a direct influence on health behaviors but also mediates the relationship between knowledge of diabetes and behavioral outcomes. This suggests, on one hand, that individuals with higher self-efficacy—those with strong confidence in their ability to perform specific future behaviors such as smoking cessation, alcohol moderation, and physical exercise—are more likely to maintain positive health cognition and consequently adopt healthier lifestyles (Ye, 2010). On the other hand, greater disease-related knowledge enhances one’s understanding of health conditions, which in turn strengthens perceived competence in preventing and managing disease, thereby facilitating the adoption and maintenance of health-promoting behaviors (Hall et al., 2016).

Furthermore, self-efficacy beliefs have been demonstrated to serve a protective function in mental health promotion (Bandura, 2004) and to mitigate the development of psychological difficulties (Givertz and Segrin, 2014; Maddux, 1995; Salami, 2010). Scheier and Carver (1985) characterized self-efficacy as a generalized expectancy for positive outcomes even in the face of adversity. For instance, high levels of self-efficacy are associated with improved regulation of stress responses, elevated self-esteem, greater psychological well-being, better physical health, as well as enhanced adaptation to and recovery from both acute and chronic medical conditions (Bandura, 1997; Hamzah et al., 2021; Mikkelsen et al., 2020). Conversely, low self-efficacy is linked to increased symptoms of anxiety and depression (Chang et al., 2018; Li, 2018), in addition to reduced subjective well-being (Jin et al., 2020; Ngui and Lay, 2020).

In early formulations of social cognitive theory, self-efficacy beliefs were conceptualized as highly contextualized knowledge structures that governed evaluation processes and subsequently guided individual behavior (Bandura, 1997). Later, Steca et al. (2009) investigated how these beliefs moderate both negative and positive affective experiences, and how they interact with supportive relationships with parents and peers to influence life satisfaction. Although limited research has directly examined the relationship between social media use for information acquisition and self-efficacy, existing studies suggest that such usage can significantly enhance self-efficacy (Kot et al., 2021; Mahmood et al., 2021; Niu et al., 2021). Specifically, the use of social media for information acquisition contributes directly to improved health knowledge—a form of informational support—which in turn can strengthen self-efficacy (Liu et al., 2019). Moreover, social media use facilitates access to diverse forms of social support (e.g., emotional companionship, self-esteem reinforcement), all of which may further promote self-efficacy (Lee and Cho, 2019).

Based on the above literature, we proposed the following hypotheses:

*Hypothesis 2*: Health knowledge mediates the relationship between social media use for information acquisition and life satisfaction.

*Hypothesis 3*: Self-efficacy mediates the relationship between social media use for information acquisition and life satisfaction.

*Hypothesis 4*: Health knowledge and self-efficacy sequentially mediate the relationship between information-acquiring social media use and life satisfaction.

## Methods

### Data

Participants were recruited through Sojump, one of the largest online survey platforms in China, which maintains a user base of approximately 2.6 million across mainland China. A total of 1,651 individuals responded to the survey invitation via email and participated in the study. Each participant received a small monetary compensation upon completion of the questionnaire. Data cleaning was conducted using two attention-check items and by screening for duplicate IP addresses, resulting in a final valid sample of 1,513 cases and an effective response rate of 91.64%.

Of the 1,513 participants, the mean age was 31.38 (from 18 to 71, SD = 9.21). They were 49.6% women (*n* = 750). Most of the participants had a bachelor’s degree (*n* = 1,050, 69.4%), and the majority of respondents were married (*n* = 959, 63.4%). The majority of monthly household income was between CNY 10,001 and 15,000 (*n* = 320, 21.2%). There were 196 participants (13%) living in rural areas and 1,317 participants (87%) living in urban areas. The average status of the participants’ mental diseases was 0.91 (SD = 1.15).

### Measurement

#### Social media use for information acquisition

Social media use for information acquisition consisted of seven items. We referred to previous research (Lin and Ho, 2018) and designed the scale based on the Chinese context. We asked respondents “How often do you get information about COVID-19 through the following channels?” and included as responses portal websites (e.g., NetEase and Tencent) social networking (e.g., Weibo and WeChat), news apps (e.g., People’s Daily and Toutiao), video sharing (e.g., Douyin and Pear Video), online Q&A communities (e.g., Zhihu), web search engines (e.g., Baidu), and online learning platform (e.g., Learning Power). Respondents were asked to respond on a five-point scale (1 = never, 5 = very often), resulting in *M* = 3.48, SD = 0.67, and Cronbach’s *α* = 0.67.

#### Health knowledge

Health knowledge consisted of both perceived knowledge and actual knowledge (Zhong et al., 2020). Of these, perceived knowledge was measured using two items related to COVID-19: “The symptoms of COVID-19 are mainly fever, fatigue, dry cough at first, and gradually difficulty breathing” and “The quarantine period for close contacts of confirmed cases is typically 14 days.” Respondents were asked to respond on a five-point scale (1 = very unknown, 5 = very well known), *M* = 4.52, SD = 0.53, and Pearson’s r = 0.30. Actual knowledge was measured by five items, using a representative statement: “The elderly and those with underlying diseases are prone to become critically ill patients” (0 = wrong, 1 = right). The total score range was 0–5 points, *M* = 4.57, SD = 0.64.

#### Self-efficacy for health management

Self-efficacy consisted of six items (Welch and Ellis, 2018). We asked respondents, “Overall, how confident are you about your ability to take good care of your health?” (e.g., “I can manage my health well” and “I can detect health risks in a timely manner”). Respondents were asked to answer on a five-point scale (1 = low ability, 5 = high ability), *M* = 3.79, SD = 0.58, and Cronbach’s *α* = 0.72.

#### Life satisfaction

Life satisfaction consisted of five items (Oh et al., 2014). We asked respondents, “Do you agree with the following descriptions of your life status?” (e.g., “My current living conditions are good” and “I am very satisfied with my current living conditions”). Respondents were asked to answer on a five-point scale (1 = strongly disagree, 5 = strongly agree), *M* = 3.12, SD = 0.86, and Cronbach’s *α* = 0.86.

#### Control variables

Age, gender, education level, marital status, monthly family income, urban/rural residence, and mental health status were used as control variables.

### Analysis

In this study, SPSS 22.0 was used to perform descriptive statistics and correlation analysis on the data. At the same time, the SPSS macro program PROCESS (Model 6) developed by Hayes (2017) was used to test the mediation model, and the bootstrap method was used to test the significance of the mediation effect.

## Results

Descriptive statistics of Pearson’s correlation for the study variables are shown in Table 1. It was found that age was positively related to self-efficacy (*r* = 0.13, *p* < 0.001) and life satisfaction (*r* = 0.18, *p* < 0.001) but negatively related to actual knowledge (*r* = −0.08, *p* < 0.01). In addition, education level was positively related to actual knowledge (*r* = 0.16, *p* < 0.001), perceived knowledge (*r* = 0.07, *p* < 0.01), and self-efficacy (*r* = 0.12, *p* < 0.001). Monthly family income was positively related to actual knowledge (*r* = 0.05, *p* < 0.05), self-efficacy (*r* = 0.20, *p* < 0.001), and life satisfaction (*r* = 0.17, *p* < 0.001). Men reported significantly higher levels of self-efficacy (*M* = 3.83, SD = 0.57) than women (*M* = 3.76, SD = 0.58), *t* = −2.36, *p* < 0.05. However, women reported significantly higher higher levels of perceived knowledge (*M* = 4.55, SD = 0.49) than men (*M* = 4.49, SD = 0.56), *t* = 1.98, *p* < 0.05.

**Table 1:** Pearson’s correlation matrix of the variables in the present study.

	1	2	3	4	5	6	7	8	9	10	11	12
1. Age	1											
2. Gender	0.15***	1										
3. Education level	−0.18***	−0.02	1									
4. Marital status	0.58***	0.06**	−0.05	1								
5. Monthly family income	0.27***	0.09***	0.32***	0.31***	1							
6. Urban/rural residence	0.21***	0.04	0.18***	0.20***	0.34***	1						
7. Mental diseases	−0.12***	−0.04	−0.01	−0.04	−0.04	−0.07**	1					
8. Social media use for IA	−0.05	0.00	0.06*	0.05	0.02	0.06*	−0.03	1				
9. Actual knowledge	−0.08**	0.02	0.16***	−0.07**	0.05*	0.06*	−0.01	−0.02	1			
10. Perceived knowledge	0.05	−0.05*	0.07**	0.02	0.04	0.05	−0.12***	0.11***	0.10***	1		
11. Self-efficacy	0.13***	0.06*	0.12***	0.17***	0.20***	0.16***	−0.19***	0.24***	−0.04	0.20***	1	
12. Life satisfaction	0.18***	0.00	0.04	0.18***	0.17***	0.17***	−0.17***	0.14***	−0.06*	0.06*	0.41***	1

IA refers to information acquisition. ^∗^*p* < 0.05. ^∗∗^*p* < 0.01. ^∗∗∗^*p* < 0.001.

### Hypotheses testing

**Figure 1:**
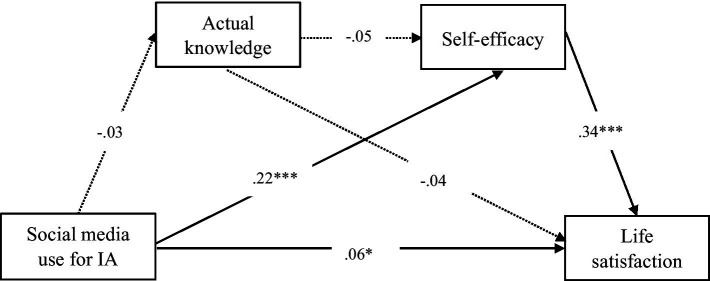
The model of social media use for information acquisition on life satisfaction for actual knowledge (*N* = 1,513). IA refers to information acquisition. The mediation roles were actual knowledge and self-efficacy. ^∗^*p* < 0.05. ^∗∗^*p* < 0.01. ^∗∗∗^*p* < 0.001.

**Figure 2:**
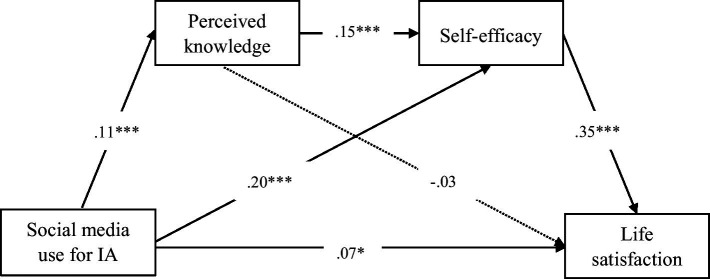
The model of social media use for information acquisition on life satisfaction for perceived knowledge (*N* = 1,513). IA refers to information acquisition. The mediation roles were perceived knowledge and self-efficacy. ^∗^*p* < 0.05. ^∗∗^*p* < 0.01. ^∗∗∗^*p* < 0.001.

Given that this study measured actual and perceived knowledge separately, we employed two distinct models using PROCESS (Model 6) to examine their respective mediating effects. The model testing the relationship between social media use for information acquisition and life satisfaction through actual knowledge was presented in Figure 1, while the model incorporating perceived knowledge was illustrated in Figure 2. Additionally, regression results predicting life satisfaction based on Model 6 were summarized in Table 2. The direct and indirect effects of social media use for information acquisition on life satisfaction were reported in Table 3.

**Table 2:** The regression on prediction of life satisfaction using Process (Model 6) (*N* = 1,513).

Predictors	Life satisfaction (for actual knowledge)			Predictors	Life satisfaction (for perceived knowledge)		
	B	SE	95% CI		B	SE	95% CI
Constant	0.55	0.27	[0.03, 1.07]	Constant	0.56	0.27	[0.03, 1.08]
Age	0.07*	0.00	[0.00, 0.01]	Age	0.07*	0.00	[0.00, 0.01]
Gender	−0.04	0.04	[−0.15, 0.01]	Gender	−0.04	0.04	[−0.15, 0.00]
Education level	−0.01	0.02	[−0.06, 0.04]	Education level	−0.01	0.02	[−0.06, 0.03]
Marital status	0.05	0.04	[−0.01, 0.16]	Marital status	0.05	0.04	[−0.01, 0.16]
Monthly family income	0.05	0.01	[−0.00, 0.05]	Monthly family income	0.05	0.01	[−0.00, 0.05]
Urban/rural residence	0.07**	0.06	[0.06, 0.31]	Urban/rural residence	0.07**	0.06	[0.05, 0.30]
Mental diseases	−0.09***	0.02	[−0.10, −0.03]	Mental diseases	−0.09***	0.02	[−0.10, −0.03]
Social media use for information acquisition	0.05*	0.03	[0.00, 0.12]	Social media use for information acquisition	0.05*	0.03	[0.01, 0.13]
Actual knowledge	−0.04	0.03	[−0.11, 0.01]	Perceived knowledge	−0.04	0.04	[−0.14, 0.01]
Self-efficacy	0.34***	0.04	[0.44, 0.58]	Self-efficacy	0.35***	0.04	[0.45, 0.60]

^∗^*p* < 0.05. ^∗∗^*p* < 0.01. ^∗∗∗^*p* < 0.001.

**Table 3:** Direct and indirect effects of social media use for information acquisition on life satisfaction (*N* = 1,513).

	For actual knowledge			For perceived knowledge		
	Effect	SE	95% CI	effect	SE	95% CI
Total effect	0.16***	0.03	[0.10, 0.22]	0.16***	0.03	[0.10, 0.22]
Direct effect	0.06*	0.03	[0.00, 0.12]	0.07*	0.03	[0.01, 0.13]
Indirect effect	0.08*	0.01	[0.06, 0.10]	0.07*	0.01	[0.05, 0.09]
SM → HK → LS	0.00	0.00	[−0.00, 0.00]	−0.00	0.00	[−0.01, 0.00]
SM → SE → LS	0.07*	0.01	[0.05, 0.10]	0.07*	0.01	[0.05, 0.09]
SM → HK → SE → LS	0.00	0.00	[−0.00, 0.00]	0.01**	0.00	[0.00, 0.01]

SM, social media use for information acquisition; HK, health knowledge; SE, self-efficacy for health management; LS, life satisfaction. ^∗^*p* < 0.05. ^∗∗^*p* < 0.01. ^∗∗∗^*p* < 0.001.

Regarding actual knowledge, the results demonstrated a positive association between social media use for information acquisition and life satisfaction (*β* = 0.06, *p* < 0.05), thus supporting H1. However, actual knowledge did not serve as a significant mediator in this relationship [*β* = 0.00, *p* > 0.05, 95% CI (−0.00, 0.00)]. Therefore, H2 was not supported. In contrast, self-efficacy significantly mediated the effect of social media use for information acquisition on life satisfaction [*β* = 0.07, *p* < 0.05, 95% CI (0.05, 0.10)], supporting H3. Finally, the sequential mediation effect through actual knowledge and self-efficacy was not statistically significant [*β* = 0.00, *p* > 0.05, 95% CI (−0.00, 0.00)]. Hence, H4 was not supported.

Regarding perceived knowledge, the results demonstrated that social media use for information acquisition was positively associated with life satisfaction (*β* = 0.07, *p* < 0.05), supporting H1. However, perceived knowledge did not mediate the relationship between social media use for information acquisition and life satisfaction [*β* = −0.00, *p* > 0.05, 95% CI (−0.01, 0.00)]. Therefore, H2 was not supported. In addition, self-efficacy significantly mediated this relationship [*β* = 0.07, *p* < 0.05, 95% CI (0.05, 0.09)], supporting H3. Finally, perceived knowledge and self-efficacy sequentially mediated the effect of social media use for information acquisition on life satisfaction [*β* = 0.01, *p* < 0.01, 95% CI (0.00, 0.01)]. Thus, H4 was supported.

## Discussion

This study examines the mechanism through which social media use for information acquisition affects life satisfaction by testing a theoretical framework. An online survey was conducted to empirically investigate this relationship. Given the prominence of COVID-19 during the research period, the study focused on this context to query participants about their social media use for information acquisition and health knowledge. The findings reveal that social media use for information acquisition directly and positively influences life satisfaction, and also affects it indirectly through the sequential mediation of health knowledge and self-efficacy. However, only perceived knowledge significantly mediated the relationship between social media use for information acquisition and life satisfaction, whereas actual knowledge did not. In addition, self-efficacy significantly mediated this relationship both in conjunction with perceived knowledge and with actual knowledge.

First, the use of social media for information acquisition was found to be positively associated with life satisfaction. This aligns with previous studies that distinguish between different types of internet and social media use—such as informational, recreational, communicative, and entertainment-related purposes—which have been shown to correlate either positively or negatively with psychological well-being depending on the specific motivation (e.g., Raacke and Bonds-Raacke, 2008; Shah et al., 2001). However, as research increasingly conceptualizes social media usage in terms of diverse audiences, varied motivations, and heterogeneous experiences—rather than simply time spent on platforms—it has revealed more nuanced effects on individuals’ social capital (e.g., Nie, 2001; Nie and Hillygus, 2002; Valenzuela et al., 2009). The present study reinforces earlier findings that certain uses of social media, including social and recreational purposes, positively influence subjective well-being. Our contribution lies in specifically examining the role of information acquisition through social media in shaping life satisfaction. It should be noted, however, that this effect may be comparatively smaller than that of social media use for social purposes (Chan, 2015).

We speculate that this may be attributed to the fact that social functions—as a core attribute of social media—and users’ social needs may hold greater significance for most users. Furthermore, specialized social media platforms, such as health applications, might be more effective in delivering health-related information and knowledge. On the other hand, a body of research suggests that social media use can also lead to misinformation, information overload, social media fatigue, and algorithmic biases (Bermes, 2021; Lee et al., 2016), all of which may negatively affect individuals’ psychological well-being. For instance, perceived information overload has been shown to indirectly predict emotional stress and social anxiety through the mediating role of social media fatigue (Pang, 2021). Additionally, information quality (e.g., accuracy or misleading content) may moderate the relationship between social media use and health knowledge. These complexities underscore the need for future research to examine different types of social media and their varied uses. More importantly, the small effect size observed for information acquisition suggests that the relationship between social media use and life satisfaction is multifaceted and warrants deeper theoretical and empirical investigation.

Second, consistent with the KAP framework, health knowledge and self-efficacy sequentially mediate the relationship between social media use for information acquisition and life satisfaction. This indicates that social media, as an emerging medium, holds significant potential for promoting individuals’ health knowledge. By applying the KAP model to social media contexts, this study extends the theoretical scope of the framework. Furthermore, the results reaffirm that self-efficacy—a well-established psychological construct—exerts strong predictive power over subjective well-being. This is consistent with prior findings indicating that the association between self-efficacy and mental health outcomes is more substantial than that of factors such as disease severity, knowledge level, or preventive behaviors (Yıldırım and Güler, 2022). The present study contributes to the literature by expanding the application of the KAP theory into the domain of subjective well-being, with particular emphasis on the role of knowledge. While previous research has primarily focused on health behavioral outcomes, this study demonstrates that the KAP framework can also effectively predict well-being, specifically life satisfaction.

To our surprise, while perceived knowledge mediated the relationship between social media use for information acquisition and self-efficacy, actual knowledge did not demonstrate a significant mediating effect. This finding aligns with previous studies highlighting the distinct roles of actual and perceived knowledge. For instance, Rimal (2000) showed that only perceived knowledge significantly predicts health behaviors, whereas actual knowledge does not. Just as life satisfaction constitutes a subjective evaluation, its predictors are also more closely linked to subjective perceptions. Although a considerable amount of information on social media may be unreliable, users who engage more extensively with such platforms tend to perceive themselves as having gained greater health knowledge. This enhanced perception, in turn, significantly strengthens their self-efficacy in managing health, ultimately contributing to higher life satisfaction. These results underscore the critical role of subjective factors in shaping life satisfaction, suggesting that perceived competence and confidence may matter more than objective knowledge in mediating the psychological benefits derived from social media use for information acquisition.

Furthermore, this study contributes to the existing literature by extending the theoretical understanding of social media-based information acquisition, highlighting the distinct role of perceived—rather than actual—knowledge in shaping psychological outcomes. Additionally, the results demonstrate that self-efficacy, whether combined with perceived or actual knowledge, serves as a significant mediator between information acquisition on social media and life satisfaction. This finding aligns with prior research indicating that only perceived knowledge significantly predicts self-efficacy, while actual knowledge does not (Johnson, 2014; Kim and Suh, 2018; Wilson, 1997). Moreover, by examining the sequential mediation of health knowledge and self-efficacy, this study reveals that neither actual nor perceived knowledge exhibits a direct relationship with life satisfaction. Instead, their influence is fully mediated through self-efficacy. These insights deepen the current understanding of the two forms of health knowledge within social media contexts and underscore the importance of differentiating between subjective and objective knowledge in digital health communication research.

### Practical implications

First, the use of social media for information acquisition primarily enhances perceived knowledge rather than actual knowledge. This underscores the context-dependent effectiveness of social media in elevating users’ subjective sense of understanding. Given the mediating role of perceived knowledge between information acquisition and life satisfaction, developers should consider integrating features designed to strengthen users’ confidence in their health knowledge. Such features could include recognition mechanisms—e.g., achievement badges or progress indicators in health literacy—as well as social reinforcement through peer interactions (such as likes and shares) to encourage continued engagement with health-related content.

However, these findings also imply that social media may create an illusion of competence by inflating perceived knowledge without substantively improving actual understanding. Therefore, health interventions should complement social media strategies with structured health education, expert-led training, or specialized platforms focused on nutrition and wellness to foster both accurate knowledge and informed behaviors.

Third, self-efficacy consistently served as a mediator across both models and demonstrated a strong association with life satisfaction, indicating that it is a more consistent and powerful predictor than health knowledge. Health communication practitioners and policymakers should prioritize the cultivation of self-efficacy—both through online social environments and offline community support—to promote sustainable improvements in life satisfaction.

### Limitations and future direction

Several limitations should be considered when interpreting the findings of this study. First, the research focused specifically on the mechanisms linking social media use for information acquisition to life satisfaction, without accounting for the potential simultaneous influence of other usage purposes (e.g., social, entertainment, or self-presentation). Moreover, different types of specialized social media platforms (e.g., health-focused vs. general social networks) may exert distinct effects on life satisfaction. Future studies should adopt a comparative approach to examine how various usage motivations—as well as different platform types and content formats—differentially affect life satisfaction.

Second, although the current study applied the KAP framework to propose a sequential mediation model, other theoretical mechanisms may offer complementary explanations. Future research could test alternative models—such as those incorporating additional cognitive, affective, or social mediators—in diverse social media contexts to develop a more comprehensive understanding of how digital information behavior influences well-being.

Third, the cross-sectional design limits the ability to establish causal or directional relationships between variables. The use of self-reported online surveys may also introduce biases such as recall bias and social desirability bias. Furthermore, as the sample was drawn from Sojump’s user base, it may overrepresent urban, highly educated populations, thereby limiting the generalizability of the results to broader demographic groups. In addition, given the diversity of social media platforms in China (e.g., WeChat vs. Zhihu), different platforms may exert distinct effects on user outcomes (Wu et al., 2025). To capture a broader overview of social media usage, this study measured engagement across multiple platform types. It should be noted, however, that this measurement approach does not constitute a rigorously validated psychological scale and should be interpreted with caution due to its relatively lower reliability.

Fourth, prior research has distinguished between two types of information acquisition behaviors: active information seeking and passive information scanning (Niederdeppe et al., 2007; Shim et al., 2006). Moreover, information acquisition may serve not only informational purposes but also social functions (O'Brien et al., 2017). Therefore, future studies should examine whether active seeking versus passive scanning of information on social media differentially influences life satisfaction. It would also be valuable to investigate how using social media for multiple purposes—such as simultaneously for information, social interaction, and entertainment—may interact to affect life satisfaction.

Finally, the focus on COVID-19—a highly salient topic during the data collection period—may have facilitated respondents’ recall of their social media use and related behaviors. However, future research should examine whether the identified mechanisms generalize to other health contexts and well-being outcomes. Additionally, while this study utilized a Chinese sample and focused on platforms such as WeChat and Douyin, the existing literature reviewed here is largely international. Unique features of the Chinese social media environment, including state-led health campaigns and comparatively closed information ecosystems, may shape how users acquire information and experience well-being, suggesting a need for more context-sensitive theoretical frameworks.

## Conclusion

This study examined how social media use for information acquisition influences life satisfaction through a sequential mediation model based on the KAP framework. The results indicate that such use not only directly increases life satisfaction, but also indirectly enhances it through the sequential mediating effects of health knowledge and self-efficacy. A notable finding is that only perceived knowledge—not actual knowledge—significantly mediates this relationship. Furthermore, self-efficacy consistently serves as a mediator, whether coupled with perceived or actual knowledge. By differentiating the roles of perceived and actual knowledge within digital environments, this study extends of the knowledge, beliefs, and practices theory and provides practical insights for the design of health communication initiatives via social media.

## Data Availability

The raw data supporting the conclusions of this article will be made available by the authors, without undue reservation.
